# Effect of ultrasonic treatment on rheological and emulsifying properties of sugar beet pectin

**DOI:** 10.1002/fsn3.1722

**Published:** 2020-06-25

**Authors:** Yang Yang, Dongdong Chen, Yang Yu, Xin Huang

**Affiliations:** ^1^ Chinese Academy of Inspection and Quarantine Beijing China; ^2^ Institute of Environment and Sustainable Development in Agriculture Chinese Academy of Agricultural Sciences Beijing China; ^3^ National Engineering Laboratory for Crop Efficient Water Use and Disaster Mitigation Key Laboratory of Dryland Agriculture and Key Laboratory for Prevention and Control of Residual Pollution in Agricultural Film Ministry of Agriculture Beijing China

**Keywords:** emulsifying property, rheological property, sugar beet pectin, ultrasonic time

## Abstract

The effects of ultrasonic treatment on rheological and emulsifying properties of sugar beet pectin were studied. Results indicated that intrinsic viscosity ([*η*]) and viscosity average molecular weight ([*M*_v_]) decreased with the increased time from 0 to 30 min but increased when the duration prolonged to 45 min. The change of apparent viscosity with shear rate of all pectin solutions could be well described by Sisko model (*R* ^2^ ≥ .996) and the infinite‐rate viscosity (*η*_∞_) and the consistency coefficient (*k*_s_) values decreased after ultrasonic treatment. Ultrasonic treatment could have an effect on dynamic moduli and activation energy of sugar beet pectin solutions. Particle size of pectin emulsions decreased and absolute zeta potential increased with increased time from 0 to 20 min. Excessive ultrasonic duration (30 and 45 min) could result in the aggregation of oil droplets in pectin emulsion and decrease in emulsifying stability. It could be concluded that ultrasonic treatment could affect the rheological and emulsifying properties of sugar beet pectin. The results have important implications for understanding the ultrasonic modification of sugar beet pectin.

## Graphical abstract

Different processing methods yielded bean sauces more distinguishable by appearance, taste, and mouth‐feel than by aroma, flavor, and after‐taste. Sauces that were brown in color, with burnt aroma and burnt after‐taste were preferred to those that were described as lumpy with mashed potato flavor. Oven roasted beans and boiled beans were preferred to traditionally roasted, extruded, and unprocessed beans.

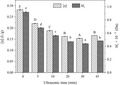

## 1 INTRODUCTION

Pectin is a complicated heteropolysaccharide extractive from cell wall materials in fruits or vegetables and mainly constitutive of a backbone α‐D‐(1‐4)‐galacturonan regions, which could be widely applied in food industry for gelling agent, stabilizer, and emulsifier (Kalapathy & Proctor, 2001; Maskey, Dhakal, Pradhananga, & Shrestha, 2018; Xu et al., 2014). Citrus peel and apple pomace can be used for pectin production for commercial use (Arioui, Ait Saada, & Cheriguene, 2017). As a by‐product of sugar beet refining industry, sugar beet pulp is also rich in pectin component which could be considered as an emerging source for pectin extraction (Li, Jia, Wei, & Liu, 2012). Sugar beet pectin exhibits good surface‐active and emulsifying properties due to the existence of high‐protein content and acetyl groups (Ma et al., 2013). The structure of pectin could be modified using the techniques such as substitution, chain extension, and depolymerization, which in turn affects its physicochemical and functional properties (Chen, Liu, Liu, Li, & Luo, 2014). Several researches have focused on property modification of sugar beet pectin involving chemical and enzymatic methods. Structural characteristic of sugar beet pulp pectic polysaccharides was changed by modification of glycanases (Oosterveld, Beldman, & Voragen, 2002). Enzymatically modified sugar beet pectin crosslinked with ferulic acid group revealed improved emulsifying stability (Zhang, Shi, et al., 2015). The emulsifying characteristic of sugar beet pectin could be modified after various enzyme treatments (Funami et al., 2011). However, chemical and enzymatic methods have many shortcomings, such as excessive time consumption, environmental pollutions, expensive cost, and complex procedures (Zhang, Zhang, Liu, Ding, & Ye, 2015). Alternative sustainable techniques should be applied in the modification of pectin.

Ultrasound is the applied science and technology of sound waves with frequency above human hearing ability ranged from 20 kHz to 10 MHz (Ma, Yang, Zhao, & Guo, 2018; Sattar et al., 2019). During processing, ultrasonication can create localized high temperature and pressure spots which could be affected by factors, such as ultrasound frequency, power intensity, temperature, and treatment time (Wang et al., 2018). As an emerging and green technology, ultrasound could be used for extraction and modification of products in food industry with relatively easy, cheap, and energy saving (Awad, Moharram, Shaltout, Asker, & Youssef, 2012). Recently, ultrasound is a promising alternative method to apply in assisted extraction of pectin from different sources compared with conventional extraction process (Bayar et al., 2017; Chen, Fu, & Luo, 2015; Maran & Priya, 2015). High efficiency of ultrasonic assisted extraction could contribute to achieving in less processing time, lower extraction temperature, and reduced energy consumption. Meanwhile, ultrasonic treatment can influence the physicochemical property, antioxidant activity, and structure of pectin in an aqueous system. It has been demonstrated that ultrasound decreased average molecular weight, changed the methylation degree, and degraded the neutral sugar side chains of citrus pectin (Zhang, Ye, Xue, et al., 2013). Intermolecular and intramolecular hydrogen bonds of citrus pectin were destructed during ultrasonic processing (Qiu, Cai, Wang, & Yan, 2019). Ultrasound also changed the rheological property and structure of apple pectin, which suggested that ultrasonic processing could be a feasible alternative method for pectin modification (Zhang, Ye, Ding, et al., 2013). Emulsifying capacities of citrus pectin had been significantly improved by ultrasound treatment (Wang et al., 2020). Modified pectin showed improved properties after ultrasonic processing. However, there has been no research on influence of ultrasonic treatment on sugar beet pectin characteristics.

In the current study, the effects of ultrasonic treatment on the rheological and emulsifying properties of sugar beet pectin under different time (0–45 min) were investigated. Rheological properties were characterized by viscosity, molecular weight, and dynamic moduli. Emulsifying properties of sugar beet pectin emulsions were measured via particle size, zeta potential, emulsifying activity, and physical stability. In addition, confocal laser scanning microscope images were also obtained to get a better understanding of ultrasonic effect on sugar beet pectin.

## 2 MATERIALS AND METHODS

### 2.1 Materials

Sugar beet pectin (Food grade) was provided by Herbstreith & Fox KG. The galacturonic acid, protein content, and degree of esterification were 73.2 g/100 g, 5.6 g/100 g, and 56.5%, respectively.

### 2.2 Ultrasonic treatment

The stock solution of sugar beet pectin (20.0 g/L) was obtained by dissolving 2.0 g pectin in volume of 100 ml deionized water under constant stirring at ambient temperature for 12 hr. Ultrasonic treatment was conducted by a JY92‐IIN Ultrasonic Homogenizer (Ningbo Scientz Biotechnology Co.) equipped with a 10 mm (diameter) probe. The probe diameter, operating frequency, and output power were 10 mm, 20 kHz, and 650 W (1%‐99%), respectively. The pectin solutions in 150 ml glass beaker were placed in the noise isolating chamber, and the ultrasonic probe was installed at the fixed depth of 20 mm below the liquid surface. The pectin solutions were then sonicated for 0, 5, 10, 20, 30, and 45 min (2 s on and 1 s off period) at the power ratio of 99% and then placed in refrigerator 4°C for further analyses.

### 2.3 Rheological property of ultrasonic sugar beet pectin

Rheological property of different pectin solutions (20.0 g/L) was determined by an AR 2000 ex rheometer (TA instruments). The aluminum cone plate geometry with 1° angle, 40 mm diameter, and 27 μm gap was used and each step was performed separately.

#### 2.3.1 Intrinsic viscosity ([*η*]) and viscosity average molecular weight ([*M*_v_])

The [*η*] of ultrasonic treated sugar beet pectin with different time was calculated according to the method of Guo et al. (2012). The apparent viscosity of different pectin samples (five concentrations of 2.0, 4.0, 6.0, 8.0, and 10.0 g/L) was measured at the angular velocity of 150 rpm and temperature of 25°C for 3 min. The [*η*] of ultrasonic sugar beet pectin under different time was calculated by Martin's equations below (Arias, Yagüe, Rueda, & García Blanco, 1998):

(1) $$\ln\left(\frac{\eta_{\text{sp}}}{c}\right)=\ln\left[\eta \right]+K\left[\eta \right]c$$

(2) $$\eta_{\text{sp}}=\frac{\eta ‐\eta_{0}}{\eta_{0}}$$

where *η*_sp_ is the specific viscosity, and *η*_0_ the viscosity of deionized water (Pa·s), respectively. *K* is Martin's constant, and *c* is concentration of pectin solution (g/L).

The [*M*_v_] was determined according to the Mark–Houwink–Sakurada equation:

(3) $$\left[\eta \right]=kM_{v}^{\alpha }$$

According to the temperature and solute–solvent system, the constant *k* was 2.34 × 10^–5^ and *α* was .8224 (Kar & Arslan, 1999).

#### 2.3.2 Apparent viscosity measurement

The apparent viscosity of sugar beet pectin was determined using the steady‐state flow step with the shear rate ranged from 0.1 to 100 s^−1^ at 25°C. The apparent viscosity (*η*) as a function of shear rate (*γ*) can be fitted by the Sisko model (Mothé & Rao, 1999):

(4) $$\eta =\eta_{\infty }+k_{s}\gamma^{n‐1}$$

where *η*_∞_ is the infinite‐rate viscosity (Pa·s), *k*_s_ is the consistency coefficient of Sisko model (Pa·s*^n^*), and *n* is the flow behavior index (dimensionless).

#### 2.3.3 Frequency sweep test

The frequency sweep tests of ultrasonic sugar beet pectin were conducted with the angular frequency ranged from 0.6283 to 62.83 rad/s at the temperature of 25°C and strain of 0.5%. The frequency (*ω*) dependence of the *G′* and *G″* could be described by the following Power Law equations:

(5) $$G^{\prime }=K^{\prime }\cdot \omega^{n^{\prime }}$$

(6) $$G^{\prime \prime }=K^{\prime \prime }\cdot \omega^{n^{\prime \prime }}$$

where *K′* and *K″* are constants (Pa·s*^n^*), *n′* and *n″* are frequency exponents dimensionless (Zhu, Li, & Wang, 2019).

#### 2.3.4 Temperature ramp measurement

The apparent viscosity as a function of temperature can be used to characterize the activation energy (*E*_a_) (Wang, Wang, Li, Xue, & Mao, 2009). Temperature ramp sweep was performed with the temperature ranged from 10 to 60°C at the heating rate of 10°C/min and angular velocity of 0.1 rad/s. The *E*_a_ could be determined according to the Arrhenius equation:

(7) $$\eta_{a}=\eta_{\infty }\exp\left(\frac{E_{a}}{\text{RT}}\right)$$

where *η*_a_ and *T* are the apparent viscosity (Pa·s) and absolute temperature (K), *η*_∞_ and *R* are the frequency factor (dimensionless) and ideal gas constant (8.3145 J/mol·K).

### 2.4 Emulsifying property of ultrasonic sugar beet pectin

#### 2.4.1 Pectin emulsions preparation

The 5.0 g of corn oil (density of 840 g/L) and 100 ml of ultrasonic treated pectin solutions (concentration of 20.0 g/L) were mixed and subjected to prehomogenization process by a digital Ultra‐Turrax Homogenizer (T25, IKA) at the speed of 12,000 rpm for 2 min. The mixtures were homogenized by an AH‐100 D homogenizer (ATS Engineering Inc.) at the pressure of 50 MPa for three passes. The prepared emulsions were placed in the refrigerator at 4°C for the following analyses.

#### 2.4.2 Particle size and zeta potential

Particle size and zeta potential of sugar beet pectin emulsions were determined by a Zetasizer Nano ZS (Malvern Instruments). To avoid multiple scattering effects, different sugar beet pectin emulsions were diluted with deionized water for 900 times (30 times each) before measurement and then injected into clear disposable zeta cell. Refractive indices of oil droplet and solvent were 1.45 and 1.33, respectively. All measurements were conducted at 25°C for at least in triplicate.

#### 2.4.3 Emulsifying activity

The prepared sugar beet pectin emulsions were diluted 900 times with 1.0 g/L sodium dodecyl sulfate (SDS) and then tested the absorbance at 500 nm using a UV spectrophotometer, and the SDS solution (1.0 g/L) was used as the blank control. The turbidity (*T*) and emulsifying activity index (EAI) were calculated by the equations below (Wang, Wang, Li, Adhikari, & Shi, 2011):

(8) $$T=\frac{2.303\cdot A\cdot V}{I}$$

where *A*, *V*, and *I* are absorbance, dilution factor, and path length (0.01 m), respectively.

(9) $$\text{EAI}=\frac{2T}{ø\cdot c}$$

where *Ø* and *c* represent oil volume fraction and pectin concentration in the emulsion, respectively.

#### 2.4.4 Confocal laser scanning microscopy (CLSM) test

The morphological characteristics of emulsion droplets were determined by an FV 3000 Confocal Laser Scanner (Olympus) equipped with a UPLXAPO 60XO (1.42 numerical aperture) silicon oil immersion objective. Sugar beet pectin emulsions were stained by Nile red according to the method of Wu et al. (2019) with minor modification. Approximately 0.5 ml of emulsion and 20 μl Nile red solutions (10 mg dissolved in 10 ml ethanol) were mixed thoroughly in a test tube. To avoid the fluorescence quenching, the stained emulsion was kept in darkened before the CLSM measurements. The excitation and emission wavelengths of Nile red are 488 nm and 600 to 700 nm, respectively. The CLSM image resolution was 1,024 × 1,024 pixels, which was corresponded to viewing filed of 200 μm × 200 μm.

#### 2.4.5 Physical stability

Physical stability of sugar beet pectin emulsions was determined using an analytical centrifuge (LUMiFuge, LUM GmbH). Emulsions (420 μl) were transferred into polycarbonate rectangular synthetic cell (2 × 8 mm) and analyzed by an emitting light beam at a wavelength of 865 nm. The samples were centrifuged at a speed of 400 rpm at 25°C with a rate of 30 s interval for 2 hr.

### 2.5 Statistical analysis

Each test was conducted at least three replicates in this experiment. At least three replicates were tested for all experiments. Data analysis was performed using the statistical software SPSS 22.0 (SPSS Inc.). Duncan's multiple comparison tests were applied to determine the significance (*p* < .05).

## 3 RESULTS AND DISCUSSION

### 3.1 Intrinsic viscosity ([*η*]) and viscosity average molecular weight ([*M*_v_]) analysis

**FIGURE 1:**
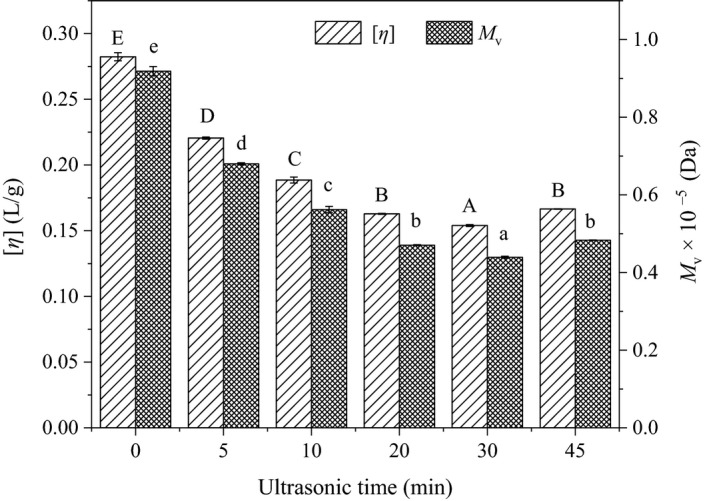
Intrinsic viscosity ([*η*]) and viscosity average molecular weight ([*M*_v_]) of sugar beet pectin under different ultrasonic time (0–45 min). Different letters on the top of columns indicate significant difference (*p* ˂ .05)

The [*η*] could directly reflect polymer solution behaviors in many applications, which is one of the most important parameters of hydrodynamic volume of given polymer mass (Rushing & Hester, 2003). The [*η*] values of sugar beet pectin solutions under different ultrasonic time from 0 to 45 min are presented in Figure 1. The [*η*] values of pectin solutions dramatically decreased from 0.282 to 0.221 L/g with the first 5 min and dropped slowly as the time ranged from 5 to 30 min. The decline of [*η*] could be attributed to the glycosidic bond breakage of pectin molecular which induced by the force of acoustic cavitation during the ultrasonic treatment (Qiu et al., 2019). The [*η*] did not continue decreasing with the increased time from 30 to 45 min but increased from 0.154 to 0.167 L/g. The increment of ultrasonic time would not increase the ultrasound effect on sugar beet pectin solutions.

As shown in Figure 1, the [*M*_v_] value decreased from 0.918 × 10^5^ to 0.439 × 10^5^ Da with the increased ultrasonic time from 0 to 30 min but increased to 0.483 × 10^5^ Da from 30 to 45 min, which was consistent with the tendency of the [*η*] change. In general, ultrasonic time increment will increase ultrasound effect but cannot increase indefinitely. Prolonged ultrasonic time had no breakage effect on the pectin chain below limiting critical size and small molecular fragments began to aggregate (Henglein, 1995). The result was consistent with the ultrasonic citrus pectin that hydrodynamic force only had destructive effects on long pectin chains above some limiting critical size (Zhang, Ye, Xue, et al., 2013).

### 3.2 Steady‐state flow analysis

**FIGURE 2:**
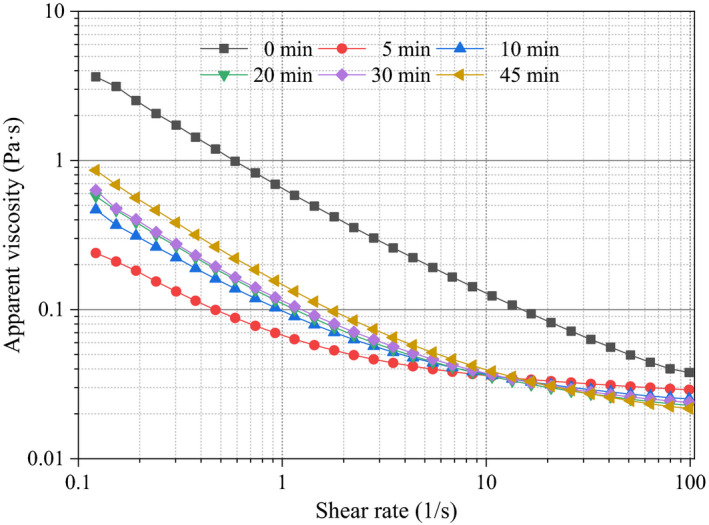
Effect of ultrasonic time on the apparent viscosity of sugar beet pectin solutions (20.0 g/L) as a function of shear rate

The apparent viscosity of ultrasonic sugar beet pectin solutions under different time is presented in Figure 2. The apparent viscosity decreased with the increasing shear rate within the test range and revealed non‐Newtonian fluid characteristic of shear thinning (Kontogiorgos, Margelou, Georgiadis, & Ritzoulis, 2012). Ultrasonic treatment under 5 min could significantly decrease the apparent viscosity of pectin solutions, which could be attributed to reduction of molecular weight caused by cavitation effect (Zhang, Ye, Xue, et al., 2013; Zheng, Zeng, Kan, & Zhang, 2018). When ultrasonic time increased from 5 to 45 min, apparent viscosity of sugar beet pectin solutions increased with the shear rate of 0.1–10 s^−1^, which could be attributed to the enhancement of pectin molecular entanglements with the increasing ultrasonic time (Hu, Chen, Wu, Zheng, & Ye, 2019). The Sisko model parameters of different pectin solutions are displayed in Table 1. The infinite‐rate viscosity (*η*_∞_) and the consistency coefficient (*k*_s_) values decreased after ultrasonic treatment but revealed no significant difference with the increasing time from 5 to 45 min (*p* ˂ .5). The flow behavior index (*n*) of Sisko model decreased from 0 to 30 min but increased from 30 to 45 min.

**TABLE 1:** Sisko model parameters of sugar beet pectin (20.0 g/L) under different ultrasonic time

Time (min)	*η*_∞_ (Pa·s)	*k*_s_ (Pa·s*^n^*)	*N*	*R* ^2^
0	0.039 ± 0.001^b^	0.607 ± 0.193^b^	0.256 ± 0.034^cc^	.999
5	0.029 ± 0.002^a^	0.047 ± 0.011^a^	0.163 ± 0.058^bc^	.996
10	0.027 ± 0.001^a^	0.070 ± 0.018^a^	0.131 ± 0.032^ab^	.998
20	0.025 ± 0.002^a^	0.083 ± 0.012^a^	0.101 ± 0.012^abb^	.999
30	0.028 ± 0.001^a^	0.081 ± 0.014^a^	0.059 ± 0.017^aaa^	.997
45	0.025 ± 0.001^a^	0.119 ± 0.004^a^	0.071 ± 0.018^ab^	.999

Note

Results were represented as mean values ± standard deviation of triplicate tests. Different letters superscripted on the results were significantly different at *p* < .05.

### 3.3 Frequency sweep analysis

**FIGURE 3:**
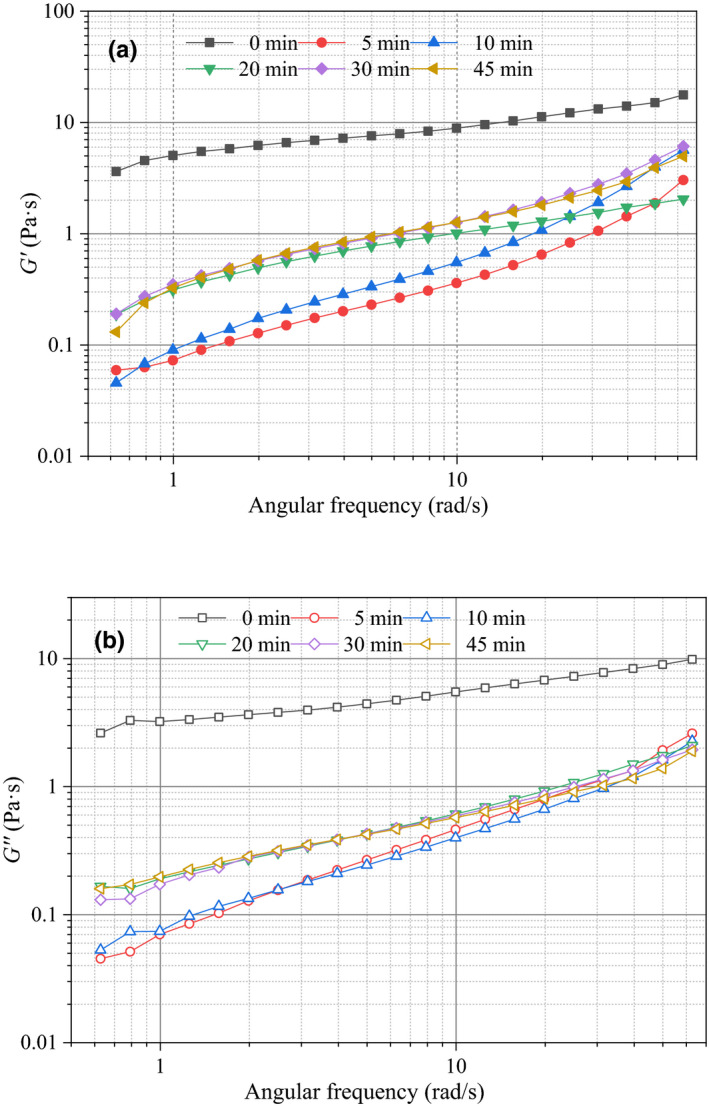
Storage modulus (*G′*) and loss modulus (*G″*) of sugar beet pectin solutions (20.0 g/L) under different ultrasonic time

The storage modulus (*G′*) and loss modulus (*G″*) of ultrasonic sugar beet pectin solutions under different time increased with the angular frequency (Figure 3). The *G′* was higher than *G″* in the measured frequency range (0.6283–62.83 rad/s). The *G′* and *G″* of pectin samples decreased as the ultrasonic time increased from 0 to 20 min. When ultrasonic time continued to increase, *G′* and *G″* did not show obvious reduction from the curves in the figures which indicated that ultrasonic treatment could influence the dynamic viscoelasticity within a certain time.

The experimental data of *G′* and *G″* could be fitted to Power Law well (*R* ^2^ ≥ .964), and the calculated parameters from the model are shown in Table 2. It could be observed that *K′* and *n′* values were greater than the *K″* and *n″* ones, demonstrating that all pectin solutions were more elastic than viscous (Alonso‐Mougán, Meijide, Jover, Rodríguez‐Núñez, & Vázquez‐Tato, 2002). The *K′* and *K″* values decreased as the ultrasonic time increased from 0 to 20 min, and the crosscurrent was observed in *n′* and *n″* values. The cavitation and mechanical effects enhanced with the increment of ultrasonic time, thus weakening the internal structure of pectin solutions (Zheng et al., 2018). As time increased from 20 to 45 min, ultrasonic treatments had no effect on the parameters which were consistent with the curves in Figure 3. The frequency dependence (*n′* and *n″)* of *G′* and *G″* for the untreated sample (0 min) had the lowest values which meant the lowest frequency sensitivity. The results showed that ultrasonic treatment could cause the viscoelasticity changes of sugar beet pectin solutions which was due to the breakage of hydrogen bonds (Xie et al., 2018).

**TABLE 2:** Power Law parameters (*K′*, *K″*, *n′*, *n″*) of sugar beet pectin solutions

Time (min)	*K′* (Pa·s*^n^*)	*n′*	*R* ^2^	*K″* (Pa·s*^n^*)	*n″*	*R* ^2^
0	4.942 ± 0.099^d^	0.267 ± 0.010^a^	.983	3.062 ± 0.061^d^	0.256 ± 0.010^a^	.981
5	1.207 ± 0.017^c^	0.286 ± 0.007^a^	.993	0.438 ± 0.006^c^	0.389 ± 0.007^b^	.997
10	0.744 ± 0.063^b^	0.346 ± 0.019^b^	.964	0.258 ± 0.008^b^	0.494 ± 0.014^c^	.991
20	0.371 ± 0.012^a^	0.544 ± 0.013^c^	.994	0.186 ± 0.003^a^	0.528 ± 0.008^d^	.998
30	0.379 ± 0.019^a^	0.526 ± 0.021^c^	.984	0.176 ± 0.010^a^	0.531 ± 0.024^d^	.980
45	0.355 ± 0.015^a^	0.547 ± 0.018^c^	.983	0.203 ± 0.002^a^	0.557 ± 0.005^d^	.982

Note

Results were represented as mean values ± standard deviation of triplicate tests. Different letters superscripted on the results were significantly different at *p* < .05.

### 3.4 Activation energy analysis

**FIGURE 4:**
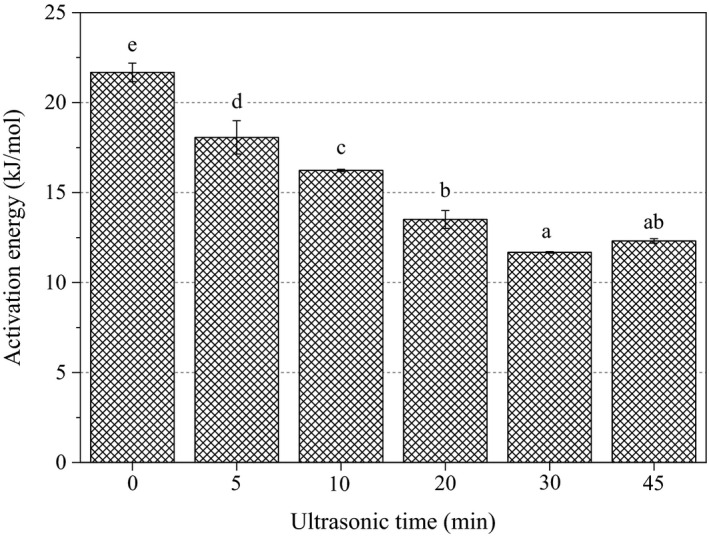
Effect of ultrasonic time on activation energy of sugar beet pectin solutions under different ultrasonic time. Different letters on the top of columns indicate significant difference (*p* ˂ .05)

The sensitivity of viscosity to temperature can be characterized by activation energy (*E_a_*) (Pongsawatmanit, Temsiripong, Ikeda, & Nishinari, 2006). The *E_a_* values of sugar beet pectin solutions under different ultrasonic time are displayed in Figure 4. The *E_a_* value decreased from 21.7 to 11.7 kJ/mol with the increment time from 0 to 30 min but increased from 30 to 45 min, which was consistent with trends in the [*η*] and [*M*_v_] as a function of ultrasonic time. The untreated sample (0 min) had the highest *E_a_* values and temperature sensitivity in viscosity, indicating that ultrasonic treatment weakened the intermolecular interactions between pectin molecules as well as intramolecular interactions between pectin polymer chains (Li, Li, Geng, Song, & Wu, 2017; Lopes Da Silva, Gonçalves, & Rao, 1994).

### 3.5 Particle size distribution, zeta potential, and emulsifying activity analysis

**FIGURE 5:**
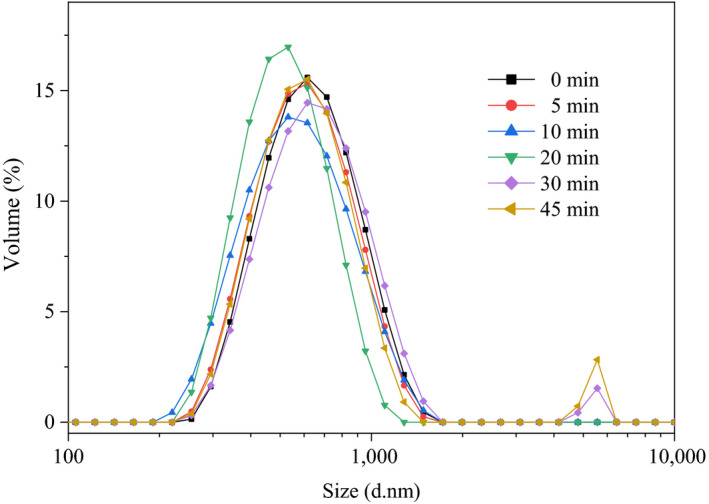
Effect of ultrasonic time on the particle size distribution of sugar beet pectin emulsions

The particle size distribution of different sugar beet pectin emulsions is shown in Figure 5. The unimodal distribution was observed in pectin emulsions with ultrasonic time from 0 to 20 min, and bimodal phenomena were detected in 30 and 45 min samples. It could be observed in Table 3 that particle size of pectin emulsions decreased as the ultrasonic time increased from 0 to 20 min and increased from 30 to 45 min, indicating that the reduction of molecular weight could promote the accessibility of surface‐active groups but too short entangled polymer chains resulted in aggregation of emulsion droplets (Alba & Kontogiorgos, 2017).

**TABLE 3:** Particle size, zeta potential, and emulsifying activity index (EAI) of sugar beet pectin (20.0 g/L) under different ultrasonic time

Time (min)	Particle size (nm)	Zeta potential (mV)	EAI (m^2^/g)
0	581.5 ± 6.7^c^	−32.4 ± 0.5^c^	73.51 ± 0.37^a^
5	549.0 ± 1.7^b^	−37.6 ± 0.2^b^	82.49 ± 0.24^c^
10	521.3 ± 4.5^a^	−37.9 ± 0.8^b^	85.53 ± 0.14^e^
20	511.9 ± 6.4^a^	−41.4 ± 0.4^a^	84.29 ± 0.14^d^
30	655.1 ± 7.0^d^	−28.8 ± 1.4^d^	73.65 ± 0.28^a^
45	704.5 ± 8.9^e^	−16.7 ± 1.8^e^	79.18 ± 0.24^b^

Note

Results were represented as mean values ± standard deviation of triplicate tests. Different letters superscripted on the results were significantly different at *p* < .05.

The zeta potential is a measure of the surface charge density which could characterize the potential stability of emulsion system and larger absolute value represents a more stable system with stronger electrostatic repulsive force (Dickinson, 2009). The zeta potential and emulsifying activity index (EAI) are presented in Table 3. It could be observed from the table that absolute zeta potential and EAI values increased first and then decreased. The 20‐min treated emulsion showed the greatest absolute zeta potential and EAI values, which meant the highest energy barrier between emulsion droplets (Sui et al., 2017). As ultrasonic time continued to increase to 30 and 45 min, the decrease in absolute values of zeta potential and EAI values was related to the aggregations of emulsion droplets, leading to a decrease in the stability and activity of different pectin emulsion. The results were in accordance with the flax seed oil emulsion (Shanmugam & Ashokkumar, 2014) and myofibrillar protein–xanthan gum emulsion (Xiong et al., 2019).

### 3.6 Microstructure of sugar beet pectin emulsions

**FIGURE 6:**
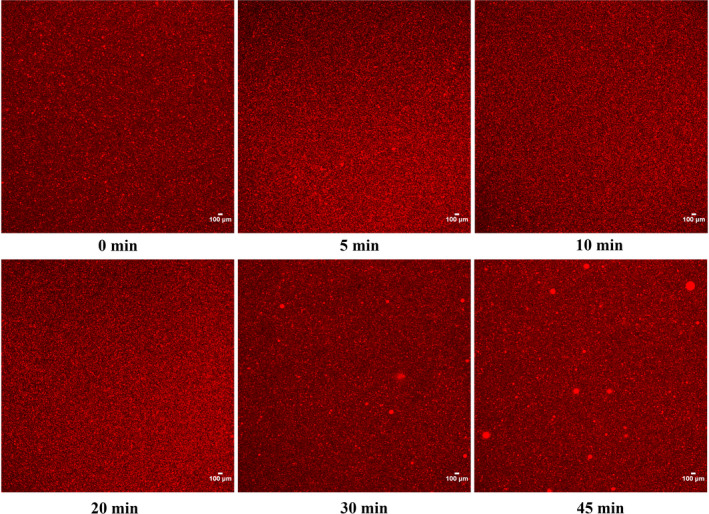
CLSM images of sugar beet pectin emulsions under different ultrasonic time

The CLSM images of different pectin emulsions are displayed in Figure 6. The bright red points represented the oil droplets stained by Nile read in pectin emulsions. The oil droplets of pectin emulsions from 0 to 20 min showed similar results with small particles and uniform size distribution. As time increased to 30 and 45 min, the observations of larger bright red zones were corresponded to aggregations of oil droplets, which further verified the particle size results in Figure 5 and Table 3.

### 3.7 Physical stability analysis

**FIGURE 7:**
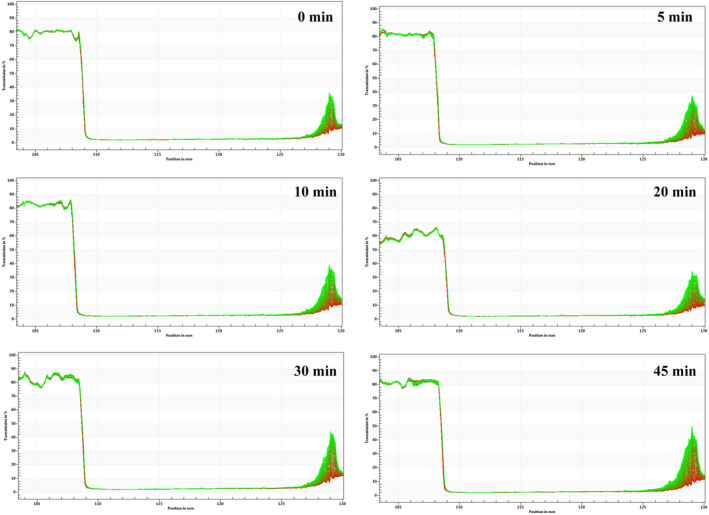
Effect of ultrasonic time on the centrifugal stability of sugar beet pectin emulsions

Macro photographs of ultrasonic pectin emulsions after centrifugation were shown in Figure 7. The abscissa is the position (mm) and ordinate is the transmission (%). Red profiles at the bottom and green ones at the top were obtained in first and last scanning. The recorded spectrum could be used to estimate the emulsion stability, and greater change of light transmittance indicates worse emulsion stability (Sobisch & Lerche, 2008; Yuan, Xu, Qi, Zhao, & Gao, 2013). It could be seen from Figure 7 that the light transmittance changes of all pectin emulsions showed similar tendency and no obvious phase separation was observed in the macro photographs. For the 45‐min treated pectin emulsion treated at 45 min, the light transmission increased at the final period of centrifugal acceleration compared with other treated samples, indicating the worst storage stability among all pectin emulsions (Xiong et al., 2019).

## 4 CONCLUSIONS

The effect of ultrasonic treatment on the rheological and emulsifying properties of sugar beet pectin was investigated in our current study. The prolonging ultrasonic duration could decrease the intrinsic viscosity ([*η*]) and viscosity average molecular weight [*M*_v_] within a certain range from 0 to 30 min. The steady‐state behavior and dynamic viscoelasticity were changed after the ultrasonic treatment. Excessive ultrasonic duration could result in the aggregation of oil droplets in pectin emulsion and decrease in emulsifying stability. The results could provide useful information about application of ultrasound in sugar beet pectin.

## CONFLICT OF INTEREST

The authors declare no conflict of interest.

## ETHICAL APPROVAL

The authors declare that this study did not involve human or animal subjects, and human and animal testing are unnecessary in this study.

## References

[fsn31722-bib-0001] Alba, K. , & Kontogiorgos, V. (2017). Pectin at the oil‐water interface: Relationship of molecular composition and structure to functionality. Food Hydrocolloids, 68, 211–218. 10.1016/j.foodhyd.2016.07.026

[fsn31722-bib-0002] Alonso‐Mougán, M. , Meijide, F. , Jover, A. , Rodríguez‐Núñez, E. , & Vázquez‐Tato, J. (2002). Rheological behaviour of an amide pectin. Journal of Food Engineering, 55, 123–129. 10.1016/S0260-8774(02)00026-2

[fsn31722-bib-0003] Arias, C. , Yagüe, A. , Rueda, C. , & García Blanco, F. (1998). Intrinsic viscosity calculated out of single point measurements for chondroitin‐4‐sulfate and chondroitin‐6‐sulfate solutions. Biophysical Chemistry, 72, 307–312. 10.1016/S0301-4622(98)00113-6 9691272

[fsn31722-bib-0004] Arioui, F. , Ait Saada, D. , & Cheriguene, A. (2017). Physicochemical and sensory quality of yogurt incorporated with pectin from peel of *Citrus sinensis* . Food Science & Nutrition, 5, 358–364. 10.1002/fsn3.400 28265371 PMC5332253

[fsn31722-bib-0005] Awad, T. S. , Moharram, H. A. , Shaltout, O. E. , Asker, D. , & Youssef, M. M. (2012). Applications of ultrasound in analysis, processing and quality control of food: A review. Food Research International, 48, 410–427. 10.1016/j.foodres.2012.05.004

[fsn31722-bib-0006] Bayar, N. , Bouallegue, T. , Achour, M. , Kriaa, M. , Bougatef, A. , & Kammoun, R. (2017). Ultrasonic extraction of pectin from *Opuntia ficus indica* cladodes after mucilage removal: Optimization of experimental conditions and evaluation of chemical and functional properties. Food Chemistry, 235, 275–282. 10.1016/j.foodchem.2017.05.029 28554636

[fsn31722-bib-0007] Chen, H. , Fu, X. , & Luo, Z. (2015). Properties and extraction of pectin‐enriched materials from sugar beet pulp by ultrasonic‐assisted treatment combined with subcritical water. Food Chemistry, 168, 302–310. 10.1016/j.foodchem.2014.07.078 25172714

[fsn31722-bib-0008] Chen, J. , Liu, W. , Liu, C. M. , Li, T. , & Luo, S. J. (2014). Pectin modifications: A review. Critical Reviews in Food Science & Nutrition, 55, 1684–1698. 10.1080/10408398.2012.718722 24798790

[fsn31722-bib-0009] Dickinson, E. (2009). Hydrocolloids as emulsifiers and emulsion stabilizers. Food Hydrocolloids, 23, 1473–1482. 10.1016/j.foodhyd.2008.08.005

[fsn31722-bib-0010] Funami, T. , Nakauma, M. , Ishihara, S. , Tanaka, R. , Inoue, T. , & Phillips, G. O. (2011). Structural modifications of sugar beet pectin and the relationship of structure to functionality. Food Hydrocolloids, 25, 221–229. 10.1016/j.foodhyd.2009.11.017

[fsn31722-bib-0011] Guo, X. , Han, D. , Xi, H. , Rao, L. , Liao, X. , Hu, X. , & Wu, J. (2012). Extraction of pectin from navel orange peel assisted by ultra‐high pressure, microwave or traditional heating: A comparison. Carbohydrate Polymers, 88, 441–448. 10.1016/j.carbpol.2011.12.026

[fsn31722-bib-0012] Henglein, A. (1995). Chemical effects of continuous and pulsed ultrasound in aqueous solutions. Ultrasonics Sonochemistry, 2, S115–S121. 10.1016/1350-4177(95)00022-X

[fsn31722-bib-0013] Hu, W. , Chen, S. , Wu, D. , Zheng, J. , & Ye, X. (2019). Ultrasonic‐assisted citrus pectin modification in the bicarbonate‐activated hydrogen peroxide system: Chemical and microstructural analysis. Ultrasonics Sonochemistry, 58, 104576 10.1016/j.ultsonch.2019.04.036 31450350

[fsn31722-bib-0014] Kalapathy, U. , & Proctor, A. (2001). Effect of acid extraction and alcohol precipitation conditions on the yield and purity of soy hull pectin. Food Chemistry, 73, 393–396. 10.1016/S0308-8146(00)00307-1

[fsn31722-bib-0015] Kar, F. , & Arslan, N. (1999). Effect of temperature and concentration on viscosity of orange peel pectin solutions and intrinsic viscosity–molecular weight relationship. Carbohydrate Polymers, 40, 277–284. 10.1016/S0144-8617(99)00062-4

[fsn31722-bib-0016] Kontogiorgos, V. , Margelou, I. , Georgiadis, N. , & Ritzoulis, C. (2012). Rheological characterization of okra pectins. Food Hydrocolloids, 29, 356–362. 10.1016/j.foodhyd.2012.04.003

[fsn31722-bib-0017] Li, D. , Jia, X. , Wei, Z. , & Liu, Z. (2012). Box‐Behnken experimental design for investigation of microwave‐assisted extracted sugar beet pulp pectin. Carbohydrate Polymers, 88, 342–346. 10.1016/j.carbpol.2011.12.017

[fsn31722-bib-0018] Li, J. , Li, B. , Geng, P. , Song, A. , & Wu, J. (2017). Ultrasonic degradation kinetics and rheological profiles of a food polysaccharide (konjac glucomannan) in water. Food Hydrocolloids, 70, 14–19. 10.1016/j.foodhyd.2017.03.022

[fsn31722-bib-0019] Lopes Da Silva, J. A. , Gonçalves, M. P. , & Rao, M. A. (1994). Influence of temperature on the dynamic and steady‐shear rheology of pectin dispersions. Carbohydrate Polymers, 23, 77–87. 10.1016/0144-8617(94)90031-0

[fsn31722-bib-0020] Ma, S. , Yang, X. , Zhao, C. , & Guo, M. (2018). Ultrasound‐induced changes in structural and physicochemical properties of β‐lactoglobulin. Food Science & Nutrition, 6, 1053–1064. 10.1002/fsn3.646 29983970 PMC6021715

[fsn31722-bib-0021] Ma, S. , Yu, S. , Zheng, X. , Wang, X. , Bao, Q. , & Guo, X. (2013). Extraction, characterization and spontaneous emulsifying properties of pectin from sugar beet pulp. Carbohydrate Polymers, 98, 750–753. 10.1016/j.carbpol.2013.06.042 23987408

[fsn31722-bib-0022] Maran, J. P. , & Priya, B. (2015). Ultrasound‐assisted extraction of pectin from sisal waste. Carbohydrate Polymers, 115, 732–738. 10.1016/j.carbpol.2014.07.058 25439955

[fsn31722-bib-0023] Maskey, B. , Dhakal, D. , Pradhananga, M. , & Shrestha, N. K. (2018). Extraction and process optimization of *bael* fruit pectin. Food Science & Nutrition, 6, 1927–1932. 10.1002/fsn3.761 30349682 PMC6189606

[fsn31722-bib-0024] Mothé, C. G. , & Rao, M. A. (1999). Rheological behavior of aqueous dispersions of cashew gum and gum arabic: Effect of concentration and blending. Food Hydrocolloids, 13, 501–506. 10.1016/S0268-005X(99)00035-1

[fsn31722-bib-0025] Oosterveld, A. , Beldman, G. , & Voragen, A. G. J. (2002). Enzymatic modification of pectic polysaccharides obtained from sugar beet pulp. Carbohydrate Polymers, 48, 73–81. 10.1016/S0144-8617(01)00216-8

[fsn31722-bib-0026] Pongsawatmanit, R. , Temsiripong, T. , Ikeda, S. , & Nishinari, K. (2006). Influence of tamarind seed xyloglucan on rheological properties and thermal stability of tapioca starch. Journal of Food Engineering, 77, 41–50. 10.1016/j.jfoodeng.2005.06.017

[fsn31722-bib-0027] Qiu, W. , Cai, W. , Wang, M. , & Yan, J. (2019). Effect of ultrasonic intensity on the conformational changes in citrus pectin under ultrasonic processing. Food Chemistry, 297, 125021 10.1016/j.foodchem.2019.125021 31253338

[fsn31722-bib-0028] Rushing, T. S. , & Hester, R. D. (2003). Intrinsic viscosity dependence on polymer molecular weight and fluid temperature. Journal of Applied Polymer Science, 89, 2831–2835. 10.1002/app.12455

[fsn31722-bib-0029] Sattar, S. , Imran, M. , Mushtaq, Z. , Ahmad, M. H. , Holmes, M. , Maycock, J. , … Muhammad, N. (2019). Functional quality of optimized peach‐based beverage developed by application of ultrasonic processing. Food Science & Nutrition, 7, 3692–3699. 10.1002/fsn3.1227 31763018 PMC6848818

[fsn31722-bib-0030] Shanmugam, A. , & Ashokkumar, M. (2014). Ultrasonic preparation of stable flax seed oil emulsions in dairy systems – Physicochemical characterization. Food Hydrocolloids, 39, 151–162. 10.1016/j.foodhyd.2014.01.006

[fsn31722-bib-0031] Sobisch, T. , & Lerche, D. (2008). Thickener performance traced by multisample analytical centrifugation. Colloids and Surfaces A: Physicochemical and Engineering Aspects, 331, 114–118. 10.1016/j.colsurfa.2008.05.040

[fsn31722-bib-0032] Sui, X. , Bi, S. , Qi, B. , Wang, Z. , Zhang, M. , Li, Y. , & Jiang, L. (2017). Impact of ultrasonic treatment on an emulsion system stabilized with soybean protein isolate and lecithin: Its emulsifying property and emulsion stability. Food Hydrocolloids, 63, 727–734. 10.1016/j.foodhyd.2016.10.024

[fsn31722-bib-0033] Wang, B. , Wang, L. , Li, D. , Adhikari, B. , & Shi, J. (2011). Effect of gum Arabic on stability of oil‐in‐water emulsion stabilized by flaxseed and soybean protein. Carbohydrate Polymers, 86, 343–351. 10.1016/j.carbpol.2011.04.059

[fsn31722-bib-0034] Wang, W. , Chen, W. , Zou, M. , Lv, R. , Wang, D. , Hou, F. , … Liu, D. (2018). Applications of power ultrasound in oriented modification and degradation of pectin: A review. Journal of Food Engineering, 234, 98–107. 10.1016/j.jfoodeng.2018.04.016

[fsn31722-bib-0035] Wang, W. , Feng, Y. , Chen, W. , Wang, Y. , Wilder, G. , Liu, D. , & Yin, Y. (2020). Ultrasonic modification of pectin for enhanced 2‐furfurylthiol encapsulation: Process optimization and mechanisms. Journal of the Science of Food and Agriculture, 100(1), 110–118. 10.1002/jsfa.10000 31436316

[fsn31722-bib-0036] Wang, Y. , Wang, L. , Li, D. , Xue, J. , & Mao, Z. (2009). Effects of drying methods on rheological properties of flaxseed gum. Carbohydrate Polymers, 78, 213–219. 10.1016/j.carbpol.2009.03.025

[fsn31722-bib-0037] Wu, D. , Wu, C. , Ma, W. , Wang, Z. , Yu, C. , & Du, M. (2019). Effects of ultrasound treatment on the physicochemical and emulsifying properties of proteins from scallops (*Chlamys farreri*). Food Hydrocolloids, 89, 707–714. 10.1016/j.foodhyd.2018.11.032

[fsn31722-bib-0038] Xie, F. , Zhang, W. , Lan, X. , Gong, S. , Wu, J. , & Wang, Z. (2018). Effects of high hydrostatic pressure and high pressure homogenization processing on characteristics of potato peel waste pectin. Carbohydrate Polymers, 196, 474–482. 10.1016/j.carbpol.2018.05.061 29891321

[fsn31722-bib-0039] Xiong, Y. , Li, Q. , Miao, S. , Zhang, Y. , Zheng, B. , & Zhang, L. (2019). Effect of ultrasound on physicochemical properties of emulsion stabilized by fish myofibrillar protein and xanthan gum. Innovative Food Science and Emerging Technologies, 54, 225–234. 10.1016/j.ifset.2019.04.013

[fsn31722-bib-0040] Xu, Y. , Zhang, L. , Bailina, Y. , Ge, Z. , Ding, T. , Ye, X. , & Liu, D. (2014). Effects of ultrasound and/or heating on the extraction of pectin from grapefruit peel. Journal of Food Engineering, 126, 72–81. 10.1016/j.jfoodeng.2013.11.004

[fsn31722-bib-0041] Yuan, F. , Xu, D. , Qi, X. , Zhao, J. , & Gao, Y. (2013). Impact of high hydrostatic pressure on the emulsifying properties of whey protein isolate–chitosan mixtures. Food and Bioprocess Technology, 6, 1024–1031. 10.1007/s11947-011-0745-x

[fsn31722-bib-0042] Zhang, L. , Shi, Z. , Shangguan, W. , Fang, Y. , Nishinari, K. , Phillips, G. O. , & Jiang, F. (2015). Emulsification properties of sugar beet pectin after modification with horseradish peroxidase. Food Hydrocolloids, 43, 107–113. 10.1016/j.foodhyd.2014.05.004

[fsn31722-bib-0043] Zhang, L. , Ye, X. , Ding, T. , Sun, X. , Xu, Y. , & Liu, D. (2013). Ultrasound effects on the degradation kinetics, structure and rheological properties of apple pectin. Ultrasonics Sonochemistry, 20, 222–231. 10.1016/j.ultsonch.2012.07.021 22982008

[fsn31722-bib-0044] Zhang, L. , Ye, X. , Xue, S. J. , Zhang, X. , Liu, D. , Meng, R. , & Chen, S. (2013). Effect of high‐intensity ultrasound on the physicochemical properties and nanostructure of citrus pectin. Journal of the Science of Food and Agriculture, 93, 2028–2036. 10.1002/jsfa.6011 23580459

[fsn31722-bib-0045] Zhang, L. , Zhang, X. , Liu, D. , Ding, T. , & Ye, X. (2015). Effect of degradation methods on the structural properties of citrus pectin. LWT ‐ Food Science and Technology, 61, 630–637. 10.1016/j.lwt.2014.11.002

[fsn31722-bib-0046] Zheng, J. , Zeng, R. , Kan, J. , & Zhang, F. (2018). Effects of ultrasonic treatment on gel rheological properties and gel formation of high‐methoxyl pectin. Journal of Food Engineering, 231, 83–90. 10.1016/j.jfoodeng.2018.03.009

[fsn31722-bib-0047] Zhu, Y. , Li, D. , & Wang, L. (2019). Dynamic rheological properties of peanut protein isolate and aggregation suspension and acid‐induced gel. Powder Technology, 358, 95–102. 10.1016/j.powtec.2018.08.052

